# The Emerging Role of Interleukin 1β (IL-1β) in Cancer Cachexia

**DOI:** 10.1007/s10753-021-01429-8

**Published:** 2021-04-27

**Authors:** Barry J. Laird, Donald McMillan, Richard J. E. Skipworth, Marie T. Fallon, D. Robert Paval, Iain McNeish, Iain J. Gallagher

**Affiliations:** 1 Insitute of Genetics and Cancer, University of Edinburgh, Crewe Road, EH4 2XR Edinburgh, UK; 2 Department of Surgical Sciences, Glasgow Royal Infirmary, University of Glasgow, Glasgow, UK; 3 Clinical Surgery, Royal Infirmary of Edinburgh, Edinburgh, UK; 4 Division of Cancer, Department of Surgery and Cancer, Imperial College London, London, UK; 5 Faculty of Health Sciences & Sport, University of Stirling, Stirling, UK

**Keywords:** Inflammation, Cachexia, Interleukin-1 beta

## Abstract

Treatment of cancer cachexia remains an unmet need. The host-tumour interface and the resulting sequestration of the pro-inflammatory cytokine Il-1β is critical in cachexia development. Neuroinflammation mediated *via* IL-1β through the hypothalamic pituitary axis results in increased muscle proteolysis and adipose lipolysis, thus creating a prolonged stress-like environment with loss of appetite and increased resting energy expenditure. Recent trials using a monoclonal antibody targeting IL-1β, canakinumab, have shown a potential role in lung cancer; however, a potential role of targeting IL-1β to treat cachexia in patients with lung cancer is unclear, yet the underlying pathophysiology provides a sound rationale that this may be a viable therapeutic approach.

## INTRODUCTION

Conservative estimates suggest cancer cachexia is attributable to over half of the cancer deaths worldwide [1]. Yet despite its prevalence, adverse impact on quality of life [2], and efficacy of anti-cancer therapy [3], there remains no standard of care and no licenced therapy [4]. To date, there has been a failure to progress the research agenda in cancer cachexia and recently tested novel therapies studies have failed to meet trial endpoints and thus regulatory approval [5, 6].

The starting point for much of the work in the last decade has been the international consensus publication of 2011 [7]. In this, cancer cachexia was defined as “a multifactorial syndrome defined by an ongoing loss of skeletal muscle mass (with or without loss of fat mass) that cannot be fully reversed by conventional nutritional support and leads to progressive functional impairment. The pathophysiology is characterised by a negative protein and energy balance driven by a variable combination of reduced food intake and abnormal metabolism.” This definition acknowledged the role of the systemic inflammatory response in cachexia and this has been supported in the intervening years by work showing the importance of systemic inflammatory responses in the progressive nutritional and functional decline of patients with cancer; indeed, the systemic inflammatory response can be regarded as a central tenet of cancer cachexia [4, 8–10]. Indeed, the European Society for Clinical Nutrition and Metabolism (ESPEN) suggest that cancer cachexia is synonymous with disease-related malnutrition in combination with inflammation [11, 12].

With reference to previous randomized trials in cachexia, few have characterized the systemic inflammatory response as an entry criterion or as a therapeutic target [5, 6, 13]. In contrast, there are increasing numbers of oncological trials incorporating measures of the systemic inflammatory response as stratification variables [14]. Indeed the presence of the systemic inflammatory response has been firmly established as being associated with weight loss and loss of lean mass [15–17], reduced functional status [18, 19], anorexia [20, 21], quality of life [2, 18, 22–24], and reduced survival [19, 25, 26].

It therefore follows that, with the systemic inflammatory response playing a key role in the development of cachexia, there is a need to target this response. Yet, to date, few studies have investigated anti-inflammatory treatment in patients with cancer cachexia [4, 9, 10].

## INFLAMMATION AND CANCER CACHEXIA

The cancer-associated systemic inflammatory response is recognized to be mediated by a network of inflammatory cytokines, eicosanoids, and other factors as part the tumour-host response. In patients with advanced cancer, pro-inflammatory cytokines predominate leading to an upregulation of Interleukin 1 (IL-1) and increased downstream production of IL-6 [27–31]. Therefore, downregulation of IL-1 is a logical therapeutic target for moderation of the systemic inflammatory response. Indeed, IL-1 inhibitors are established in the treatment of inflammatory joint disease.

### Role of IL-1 in Cachexia

In cachexia, the IL-1 pathway is overactive and contributes to cachexia *via* several mechanisms [32]. It influences tryptophan secretion resulting in increased concentrations and subsequently excess hypothalamic derived serotonin, causing satiety, and appetite suppression [33]. Whilst the actions of these cells normally represent an acute physiological response to stress, they are exacerbated by cancer where an inflammatory environment causes prolonged stress, resulting in overstimulation causing muscle wasting [9]. The impact of IL-1 on this mechanism has been demonstrated by pre-clinical models in which IL-1 triggered the release of α-melanocyte stimulating hormone (α-MSH) from pro-opiomelanocortin (POMC) neurones. Then, α-MSH stimulates Melanocortin-4 (MC4-R) neurones to induce appetite suppression [34]. Furthermore, IL-1 exhibits an inhibitory effect on Neuropeptide Y (NPY) neurones which also physiologically inhibits MC4-R neurones [35, 36].

Studies have also shown that IL-1 stimulates hypothalamic neurones which release corticotrophin releasing hormone (CRH) [37]. This causes secretion of adrenocorticotropic hormone and cortisol, which may in turn mediate the catabolic effects of cachexia. This link between IL-1, the hypothalamic pituitary axis (HPA) and muscle wasting was explored further by in a study by Braun et al. where inflammation was induced in mouse models *via* intracerebroventricular injections of IL-1 [38]. The mice exhibited an increase in muscle-specific E3 ubiquitin ligases, a key requirement for the catabolism of muscle. The same result was then also achieved when inflammation was induced peripherally with LPS. Interestingly however, this study showed that muscle catabolism was ameliorated in mice which had undergone an adrenalectomy. Such results infer that activation of the HPA axis is central to the catabolic effects of IL-1.

Two IL-1 genes, IL1A and IL1B, encode IL-1α and IL-1β and binding of either IL-1α or IL-1β to the IL-1R1 receptor. Although similarities exist between IL-1α and β, clear differences are also present with the latter having a greater pro-inflammatory effect, influencing carcinogenesis and invasiveness of cells [39]. The potential role of Il-1α has been recently reviewed [40].

### Role of IL-1β in Cancer

IL-1β is produced in response to inflammation by monocytes, macrophages, and neutrophils and induces production of stimulation of IL-6 [41]. IL-1β is released from macrophages and along with its potent inflammatory effect it has been widely established that IL-1β production is upregulated in ovarian, lung, and GI cancers and in general these cancers are associated with bad prognoses [42]. IL-1β gene is located on chromosome 2q14 within a 360-kb region and it has been shown that key polymorphisms, IL-1beta +3954, are a risk factor for the development of cachexia in patients with gastrointestinal cancers [42]. This finding has been supported by other work demonstrating the central role of IL-1β in cachexia [43–46].

### Role of IL-1β in Cachexia

IL-1β also plays a role cachexia development through the CNS. Stimulated *via* peripheral inflammation, central inflammation (specifically the hypothalamus) results in release of multiple inflammatory factors and alteration in neural mechanisms that result in proteolysis and lipolysis. Further, this HPA-mediated abnormal regulation causes muscle breakdown with the resulting supply of amino acid precursors for superfluous hepatic gluconeogenesis. The resulting dysregulation of POMC and AGRP [47], both of which control lean muscle proteolysis and adipose lipolysis, and MC4-R which regulates appetite and energy expenditure [48] causes muscle wasting [9]. IL-1β has been demonstrated as being a critical factor in this process and thus a key player in the loss of lean mass, appetite, and inflammatory upregulation, seen in cancer cachexia [38, 43–46].

### Targeting IL-1β in Cancer

It follows that as the pathophysiological mechanisms of cancer cachexia are mediated by IL-1β, targeting this is a logical approach. Canakinumab is a human monoclonal antibody to IL-1β with a half-life of 26 days. Binding directly to circulating IL-1β, it neutralizes activity between IL-1β and its receptor, making it well suited to a therapeutic role. Although this role has been established for over a decade in rheumatological indications and other immunological diseases, it has recently been shown to efficacious in prevention of cardiac disease, in comparison to placebo (CANTOS trial) [49]. Of particular relevance was a subsequent analysis of the CANTOS cohort where the incidence of lung malignancy was noted to be reduced in those patients taking higher doses (150 mg or 300 mg) of canakinumab [50]. This finding is perhaps not surprising as the clear role of the inflammatory response in the development of lung cancer is well established. It therefore follows that attenuation of this targeting the potent pro-inflammatory mediator IL-1β *via* canakinumab may yield benefit. Ridker and co-workers also observed that the beneficial effect was most likely in those with raised CRP. Again, this observation echo’s recent work by Dolan and co-workers highlighting the role of CRP in influencing outcomes in clinical trials of anti-cancer therapies [14, 51].

The role of Canakinumab in the inflammatory process in malignant disease is being examined; however, the pathophysiological mechanisms are plausible. Currently, the CANOPY suite of clinical trials in lung cancer is underway and findings awaited with interest. However, the potential role of canakinumab in the prevention or treatment of cancer cachexia is unclear. Survival endpoints are predominant in current trials and whilst quality of life and other patient reported outcomes are examined, key endpoints focussing on cancer cachexia are not within the remit of the current trials.

### Potential Role of Targeting IL-1β in Cancer Cachexia

Several studies further support the potential role of canakinumab in the treatment of cancer cachexia. Djamil and colleagues demonstrated that patients with cancer-related anorexia had significantly higher levels of IL-1β than comparators and that this was correlated with severity of anorexia [52]. In patients with pancreatic cancer, Fogelman and co-workers showed that IL-1β levels were predictive of development of cancer cachexia and was superior in this regard to other cytokines [53]. Another prospective study demonstrated the superiority of IL-1β over IL-6 in terms of correlation with the clinical phenotype of the cachexia syndrome [46].

Other work has echoed these findings further substantiating the clear influence of IL-1β in cancer cachexia genesis [54, 55].

## CONCLUSION

IL-1β represents a cytokine involved in the pathophysiology of cancer, with direct targeting a viable avenue to treat cancer cachexia as well as tumour load *per se*. Work to date highlights the key role of IL-1β in the central mechanisms of cancer cachexia supported by emerging clinical trials. Yet to fully explore this, trials are needed which include patients at risk of cancer cachexia and that use endpoints sensitive enough to examine the multiple clinical sequelae of cancer cachexia. Further, cancer cachexia is a complex phenomenon and targeting IL-1β in isolation may be insufficient but combined with a background standard of cachexia care, it may have its most optimal effect. Canakinumab has been shown to have excellent tolerability in multiple completed and ongoing clinical trials, as well as real world experience [56]. The next logical step is to undertake exploratory trials examining canakinumab with robust cachexia endpoints as key outcomes. Such trials are eagerly awaited.
